# The progress in our understanding of CIN in breast cancer research

**DOI:** 10.3389/fonc.2023.1067735

**Published:** 2023-02-16

**Authors:** Yu-Yang Liao, Wen-Ming Cao

**Affiliations:** ^1^ Wenzhou Medical University, Wenzhou, China; ^2^ Department of Breast Medical Oncology, Zhejiang Cancer Hospital, Hangzhou, China

**Keywords:** genetic instability, chromosomal instability, aneuploidy, breast cancer, prognosis, treatment, metastasis, drug resistance

## Abstract

Chromosomal instability (CIN) is an important marker of cancer, which is closely related to tumorigenesis, disease progression, treatment efficacy, and patient prognosis. However, due to the limitations of the currently available detection methods, its exact clinical significance remains unknown. Previous studies have demonstrated that 89% of invasive breast cancer cases possess CIN, suggesting that it has potential application in breast cancer diagnosis and treatment. In this review, we describe the two main types of CIN and discuss the associated detection methods. Subsequently, we highlight the impact of CIN in breast cancer development and progression and describe how it can influence treatment and prognosis. The goal of this review is to provide a reference on its mechanism for researchers and clinicians.

## 1 Introduction

There are two types of genetic instability (GI). The first instability occurs at the nucleotide level and is caused by mismatch-repair gene mutations. Its hallmark feature is the microsatellite instability (MIN), which can increase the frequency of point mutations or small fragment insertions/deletions. The second instability occurs at the chromosomal level, and it is defined as chromosomal instability (CIN). CIN is ubiquitous in cancer and a driving factor of tumor heterogeneity, and its existence and extent have a great impact on treatment efficacy and patient prognosis (1). Tumor cells with CIN are more likely to adapt to changes in the microenvironment, which is conducive to tumor cells escaping innate immunity, promoting metastasis, and developing drug resistance (2). CIN involves the activation of the CGAS–STING pathway, which promotes metastasis (3). Furthermore, CIN cell subsets exist in most types of breast cancer (4), and CIN is associated with poor prognosis (5). However, some studies have observed that extensive CIN makes it difficult for breast cancer cells to adapt to the tumor microenvironment, and such patients have a better prognosis (6). Therefore, the relationship between CIN and breast cancer requires further investigation.

## 2 Overview of chromosomal instability

### 2.1 Definition and types of chromosomal instability

**Figure 1:**
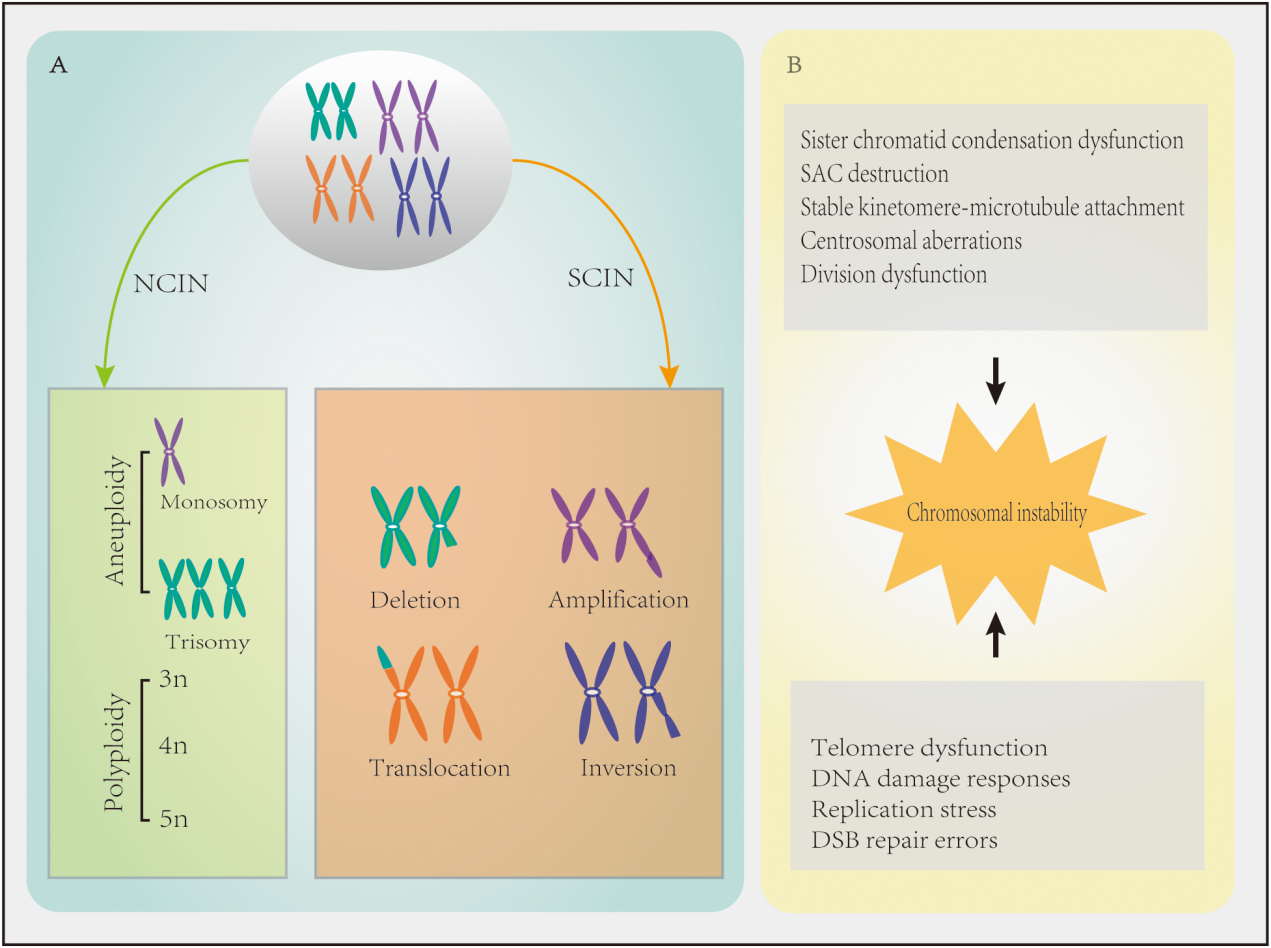
Chromosomal instability (CIN) in cancer characteristics. **(A)** Numerical chromosomal instability (NCIN) results in an abnormal chromosome number. Structural chromosomal instability (SCIN) refers to gain or loss of chromosome fragments or chromosomal rearrangements, resulting in deletions, amplifications, translocations, and/or inversions. **(B)** Mechanisms contributing to defects in CIN.

Chromosomal instability refers to the increase in the frequency of gaining or losing whole chromosomes or chromosome segments during chromosome separation (7), leading to changes in chromosome number and structure. There are two types of CIN, namely numerical chromosomal instability (NCIN) and structural chromosomal instability (SCIN) (**Figure 1A**). NCIN refers to the gain or loss of an entire chromosome or chromosome set (8), while SCIN refers to the change of chromosome segments through deletion, amplification, translocation, rearrangement, or inversion (9).

### 2.2 Mechanism of chromosomal instability

When chromosomal segregation is disrupted, NCIN usually results (10). Chromosomal mis-segregation is characterized by sister chromatid condensation dysfunction, spindle assembly checkpoint (SAC) destruction, overly stable kinetomere-microtubule attachment, centrosomal aberrations, and cell division dysfunction (11)(**Figure 1B**). The overexpression of SAC genes (e.g., *BUB1, BUB1B, CDC20, MAD1, MAD2L1, TRIP13*, and *TTK*) in breast cancer leads to checkpoint over-activation and mitotic slippage, which halts cells in mitosis and promotes entry into the G1 phase of the next cell cycle in the absence of cytokinesis and polyploidy, thereby promoting CIN (9). In addition, overexpression of *TPX2* (a mitotic regulator involved in the formation of mitotic spindles) and overexpression of *UBE2C* which can ignore the detection signal of mitotic spindles, both of them can induce CIN (12, 13). *MYC* overexpression also leads to CIN by delaying the mitotic process (14). Centrosomal aberrations, including centrosomal amplifications (CA), structural defects, and loss of primary ciliated nuclei, which all lead to CIN (15), are also common in breast cancer. For instance, CA has been reported in ≥75% of breast cancer cases (16). Many proteins that control cytokinesis in breast cancer (e.g., *AURKA, AURKB, BRCA2, CEP55, FOXM1*, and *KIF20A*) are often mutated or overexpressed, leading to cytokinesis failure and tetraploid binucleated cell formation (11).

SCIN is more likely to occur when telomere dysfunction (e.g., telomere shortening), defective DNA damage responses, replication stress, and DNA double-strand break (DSB) repair errors occur (4, 7, 11, 15) (**Figure 1B**). The faulty repair of DSBs can be caused by mutations in DNA repair genes, such as *BRCA1* and *BRCA2*, and the activation or overexpression of oncogenes, which can lead to chromosomal translocations, duplications, and deletions (17, 18). Translocation can form oncogenes in the form of fusion genes (19). For instance, the *ETV6–NTRK3* oncogene promotes the occurrence of breast cancer by activating the *AP1* complex (4). It was recently demonstrated that the *APOBEC3B* gene is highly expressed in breast ductal carcinoma in situ, which induces replication stress through incomplete DNA replication and CIN (20). In addition, the low methylation of the centromere comprised of satellite α repeats can result in the high expression of satellite α transcription factors, which increases the copy numbers of breast cancer chromosomes 8q and 20q and causes CIN (21).

Despite their apparently distinct mechanism, it is worth mentioning that both NCIN and SCIN often coexist in tumors (7). NCIN can induce SCIN and vice versa (22). In addition, the two types of CIN also have different consequences (23). For example, NCIN often leads to DNA damage and p53 activation, while SCIN often leads to chromothripsis etc, which further aggravate GI (7). In terms of drug therapy, SCIN is more sensitive to chemotherapy and prone to drug resistance to afatinib, lapatinib and austocystin D, while NCIN is more sensitive to radiotherapy and prone to drug resistance to BRAF inhibitors (22). The differences in various aspects between these two types of CIN suggest that it is necessary to refine CIN. Different research methods and treatments may be used for different types of CIN.

## 3 Methods of chromosomal instability detection

Despite the importance of CIN in breast cancer, it cannot be routinely detected in clinic due to technical shortcomings (24). Although most studies still detect CIN by traditional detection methods, such as comparative genomic hybridization (CGH), single nucleotide polymorphism (SNP) array, polymerase chain reaction (PCR), and flow cytometry, these methods are not excessively accurate. Because these methods employ cell population-based averaging to identify gene copy number changes within samples, and exactly equate these findings with CIN (25). But even so, the NCIN or SCIN score obtained by the above methods is still a fairly good proxy measures for CIN, because CIN results are often numerical or structural changes that can be easily detected by the above methods. However, many scholars are still searching for more precise detection methods. Geigl et al. (26) reported that CIN can be accurately detected by tracking the number of chromosomes in a single cell and its progeny, or quantifying the intercellular heterogeneity of chromosomal changes within a cell population at a specific time point on the premise that CIN drives karyotype heterogeneity. These methods can include live-cell or transgenic chromosome labeling approaches such as the fluorescent labeling or manipulation of chromatin-associated proteins, manipulation of reporter systems, fluorescent labeling of artificial chromosomes, and modification of gene-editing systems. Cytogenetic methods, including karyotyping, fluorescence *in situ* immunohybridization (FISH), spectral karyotyping (SKY), and multibanding (M-banding) techniques can also be employed. In addition, quantitative microscopy and cytometry can also be applied, as well as single-cell comparative genome hybridization, single-cell whole genome sequencing, and single-cell copy number variation analysis (1, 9). The advantages and disadvantages of these methods, in addition to representative examples of their clinical and research applications can be found in the review (25).

Besides, Xu et al. (24) proposed a deep learning model to detect CIN in breast cancer, which applied histopathology (i.e., hematoxylin–eosin stained sections) and genomic aneuploidy loading methods to estimate the extent of CIN. This approach combined computer technology with pathology, with a sensitivity and specificity of 81.2% and 68.7%, respectively. However, this approach only approximated the extent of CIN in some regions of the tumor and failed to provide important information on spatial heterogeneity.

Next-generation sequencing (NGS) provides a new approach for evaluating CIN. For instance, low-pass whole gene sequencing (LPWGS) (27) has been applied in the field of prenatal diagnosis. Wells et al. (28, 29) used this method to detect copy number changes in human embryonic cells, with a sensitivity and specificity of 100%. This method can reflect the changes of the whole genome with high efficiency and low cost, but the disadvantage is that high quality specimens are required. Low-pass whole gene sequencing can also be used to assess the extent of CIN in tumors by detecting plasma-free DNA. This non-invasive method allows clinicians to track chromosomal changes, and it has been recently used to study triple-negative breast cancer (TNBC) (30, 31).

In addition, there are many indirect methods to assess the extent of CIN such as micronucleus assay (32, 33), human artificial chromosome detection (34), CA20 detection (35), and CEP17 copy number determination (36).

## 4 Chromosomal instability and breast cancer cell proliferation and metastasis

Ben-David et al. (37) demonstrated that CIN-induced aneuploidy can either promote or inhibit the proliferation of breast cancer cells, but the mechanism is unknown. Santaguida et al. (38) reported that the promotion or inhibition of cell proliferation may be related to gene copy number changes, proteotoxicity induced by aneuploidy, and P53 activation caused by chromosomal mis-segregation. David et al. (39) found that copy-number amplification of core regulators, including *TPX2* and *UBE2C*, might regulate proliferation of high CIN tumors by regulating CIN-specific gene expression modules. The kinesin KIF18A regulates microtubule dynamics in spindles to enable proper chromosomal alignment. Recently, Marquis et al. (40) revealed that KIF18A may be essential for the proliferation of breast cancer cells with CIN. The knockdown of KIF18A significantly inhibited the proliferation of a breast cancer cell line, but showed no effect on the extent of CIN. These findings can be explained by the fact that the spindle microtubule polymerization rate increased and kinetochore microtubule turnover changed in cells with CIN, which may have enhanced the dependence on KIF18A function to inhibit microtubule growth. In the absence of KIF18A activity, kinetomere–microtubule attachment and centrosomal integrity are impaired, thereby resulting in mitotic prolongation and centrosomal fragmentation without the ability to complete mitosis and cell death.

Several studies have reported that the extent of CIN in metastatic breast cancer was higher than that in primary breast cancer (41), indicating that CIN is associated with metastasis (42). However, it is unknown whether CIN is a concomitant factor or a driving factor of breast cancer metastasis. Some researchers believe that CIN is a “by-product” of tumor progression, at which time tumor cells can lose or gain chromosomes, while other investigators contend that CIN is a feature of early-stage cancer that leads to the loss or inactivation of tumor suppressor genes (19). Cancer cells with CIN are more adaptable and capable of metastasizing due to extensive genetic changes (43). For instance, the loss of heterozygosity on chromosome 8p results in the downregulation of 8p localization genes (*ASAH1, FDFT1, LEPROTL1, EPHX2*, or *BNIP3L*), leading to the upregulation of genes in mevalonate and fatty acid metabolism pathways. When lipid metabolic processes are disrupted in the breast cancer cell line MCF10A, the activities of small GTPases, such as RHO, RAC, and RAS, are increased, which promotes invasion and metastasis (8, 44). The amplification of chromosome 1q21.3 is common in breast cancer, with an incidence of 10%–30% in primary lesions and more than 70% in recurrent and metastatic lesions. The amplification of this chromosome leads to aberrant gene expression, which is related to metastasis (30). Genetic changes can also promote CIN. For instance, SCIN induced by the fusion of estrogen receptor 1 (*ESR1*) with *PCDH11X* (encoding a cell adhesion protein) or *YAP1* (encoding a YES1-related transcriptional regulator) can induce epithelial-mesenchymal transition (EMT) of the breast cancer cell line T47D (45). Furthermore, *MASTL* overexpression induces CIN and promotes metastasis (46). Mutations in *TP53* (47), *PTEN* (48), and *MYC* (49) can also induce CIN, with the mutation rate in metastatic breast cancer being higher than that in primary breast cancer (42).

Bakhoum (3) et al. performed *in vivo* and *in vitro* experiments showing that CIN was the driving factor of breast cancer metastasis through the formation of micronuclei, which were caused by chromosomal segregation errors, and the breakdown of micronuclei, which induced genomic DNA entry into the cytoplasm. The activation of the cGAS–STING (a cGMP-AMP synthetase stimulator of the *IFN* gene) cytoplasmic DNA sensing pathway and downstream non-classical NF-κB signaling pathway promoted the expression of inflammatory and EMT genes required for metastasis. More importantly, the authors demonstrated that the inhibition of CIN significantly delayed metastasis, while persistent segregation errors promoted cell invasion and metastasis, thus establishing a causal link between CIN and metastasis.

In summary, CIN can lead to metastasis at multiple levels. CIN caused by gene variation occurs randomly, so there is heterogeneity within tumors. Some types of CIN can cause changes in specific genes and promote metastasis. In terms of immunology, there is no need for CIN to cause special gene variations. According to the present study, as long as thers is CIN, metastasis can be facilitated, but this is highly correlated with the extent of CIN.

## 5 Chromosomal instability and breast cancer prognosis

CIN is associated with poor prognosis of breast cancer (41). This can be explained by the fact that tumor heterogeneity caused by CIN produces subclones that are more aggressive and likely to metastasize, thereby promoting tumor progression (1). Studies have demonstrated that CIN is an independent prognostic factor for breast cancer (HR = 3.563 P = 0.005) (50). Birkbak et al. (51) conducted a retrospective analysis of 2125 breast cancer patients from 13 studies and reported that the extent of CIN of ER^-^/HER2^-^ breast cancer was significantly higher than that of ER^+^ breast cancer, and the extent of CIN of basal cell-like breast cancer was the highest (52), indicating that CIN is a manifestation of high-risk breast cancer subtypes. Goh et al. (30) observed that S100A7/8/9, which was encoded by the 1q21.3 amplification region, formed a regulatory loop with IRAK1 to drive tumor growth, chemotherapy resistance, and metastasis, which were associated with tumor recurrence and poor prognosis. In 2018, Stover et al. (31) first reported that the incidence of cfDNA CNVs in triple-negative breast cancer patients was 96.3% by LCWGS, which was significantly associated with breast cancer survival (median survival 15.9 months in the low-abundance variant group and 6.4 months in the high-abundance variant group). Copy number variations were also an independent prognostic factor (HR = 2.14, P < 0.001). Further studies showed that, compared with patients without metastases, patients with metastatic TNBC had higher amplification rates of 18q11 and 19p13, and shorter survival times than patients with amplifiable TNBC (HR = 3.3, P = 0.012). Liu et al. (53) observed that the 17q23 amplification occurred in approximately 11% of breast cancer cases, and in HER2^+^ breast cancer, the 17q23 amplification was significantly associated with poor prognosis. Lee et al. (36) examined 534 cases of invasive breast cancer and reported that patients with extensive CIN had significantly shorter disease-free survival (P = 0.002), while Mo et al. (50) used LPWGS on cfDNA specimens from 65 patients with metastatic breast cancer to obtain the CIN scores and observed that patients with extensive CIN had a shorter survival time. In addition, the amplification or overexpression of *CCND1*, *FGF4*, and *FADD* at the 11q13.3 locus was closely associated with shorter recurrence-free survival (HR = 1.16, P < 0.05; HR = 0.72, P < 0.05; HR = 1.59, P < 0.05) (54).

However, the results of other studies were contradictory to those of the above studies. Birkbak et al. (51) observed that 265 triple-negative breast cancer patients with extensive CIN had a better prognosis (P = 0.021), and as the CIN level increased, the recurrence risk decreased and the survival time increased. Jamal et al. (55) analyzed 1173 breast cancer patients enrolled in the TACT study and reported that extensive CIN was associated with better prognosis in patients with ER^-^/HER2^-^ breast cancer (P = 0.03 and 0.007, respectively), indicating that CIN is a good prognostic marker of breast cancer.

These findings indicate that there is no simple relationship between CIN and patient prognosis. Tumors with high cell proliferative and metastatic ability show a certain degree of CIN, while extensive CIN may disrupt genomic instability to the point of non-repair, which is not conducive to tumor growth.

## 6 Chromosomal instability and therapeutic efficacy

CIN has been applied to predict the sensitivity and resistance of anti-cancer drugs, such as anthracyclines and taxanes, and individualized treatments (56, 57). In 2010, Bartlett et al. (58) reported that HER2^+^ breast cancer patients with moderate CIN were more sensitive to treatment with taxanes, while patients with extensive CIN were more sensitive to treatment with anthracyclines and platinum-based drugs. Unfortunately, the mechanisms were not investigated. Vargas et al. (59) demonstrated that ER^+^/HER2^-^ breast cancer patients with moderate CIN were more sensitive to treatment with taxanes and anthracyclines than ER^-^/HER2^-^, ER^-^/HER2^+^, and ER^+^/HER2^+^ cell lines with moderate CIN, suggesting that the clinical significance of CIN in predicting treatment efficacy is different for different subtypes of breast cancer. Furthermore, the authors concluded that there was a threshold for CIN beyond which resistance or sensitivity to treatment can occur, and that this threshold depended on the status of ERα and HER2, although these findings remain to be validated in a prospective study of patients with defined tumor stage and treatment history. In 2021, a study of 131 TNBC patients who were treated with carboplatin demonstrated that patients with high CIN had a shorter PFS than patients with low and moderate CIN [3.4 months versus 5.7 months, P = 0.027] (60). Furthermore, Scribano et al. (61) observed that paclitaxel at therapeutic concentrations could induce multipolar spindle formation rather than mitotic arrest. In this study, paclitaxel promoted the multiple stages of spindle formation, thereby increasing chromosomal segregation errors in cancer cells and causing cell death. In addition, breast cancer cell lines with extensive CIN are more sensitive to paclitaxel treatment. By upregulating *MAD1* or downregulating *CENP-E*, the extent of CIN can be increased and the sensitivity of cells to paclitaxel can be improved.

CIN can improve drug sensitivity, but it can also initiate drug resistance. In 2017, Wein et al. (62) reported that CIN and the tumor heterogeneity caused by CIN were the causes of chemoresistance of TNBC. Subsequently, Lukow et al. (63) demonstrated that extensive CIN and its resulting multiple karyotypes of breast cancer cells could drive stress adaptation and promote drug resistance. Similarly, Ippolito et al. (64) observed that GI increased karyotype heterogeneity, reduced the proportion of favorable karyotypes for the survival of normal cells, and increased the proportion of favorable karyotypes for the survival of tumor cells, thereby leading to drug resistance. Other studies revealed that cancer cells may employ gene copy number changes, especially induced repetitive karyotype changes, to selectively survive chemotherapy and other stresses and to gain drug resistance. Zhou et al. (54) observed that the 11q13.3 amplification was detected in 14.6% of invasive breast cancer cases, and it was often detected in luminal B breast cancer. *CCND1*, an oncogene at the 11q13.3 locus and it’s products have been reported to drive cell proliferation, angiogenesis, and drug resistance; *CCND1*, *FGF* expression (mainly *FGF3/4/19*), and *FADD* overexpression or co-expansion, which was negatively correlated with CD4^+^ T cell number and dendritic cell infiltration, suggesting that its expansion may reduce immune activity in breast cancer cells. These results also suggest that the 11q13.3 amplification may be a key factor in the development of drug resistance in breast cancer. For instance, Mo et al. (50) reported that gene mutations on chromosomes 8 and 17 were frequent in metastatic breast cancer patients with drug resistance (P = 0.010 and 0.051, respectively), whereas gene deletions on chromosomes 9 and 7q were associated with drug resistance (P = 0.039 and 0.021, respectively). Gomes-Miragaya et al. (65) observed that breast cancer patients with TNBC/BRCA1 mutations and resistance to docetaxel often had the 12p amplification. However, it is unclear whether the 12p amplification existed before docetaxel treatment, and if it did, then the 12p amplified tumor cell subsets would have also increased after docetaxel treatment or resistance. In addition, the 12p amplification enhanced the sensitivity of tumor cells to carboplatin, and sequential use of docetaxel and carboplatin could improve the survival rate of patients with TNBC/BRCA1 mutations.

CIN cannot only improve the sensitivity of cancer cells of drugs, but also render cancer cells resistant to drugs, a phenomenon that has been observed in several studies. CIN is characterized by heterogeneity, leading to differences in study results, indicating that the relationship between the extent of CIN and the efficacy of treatment requires further investigation.

## 7 Chromosomal instability-targeted strategies for breast cancer treatment

CIN is an independent prognostic factor of breast cancer that is also related to drug sensitivity. Therefore, methods that can alter the extent of CIN are expected to become new treatment strategies for breast cancer (19). Although there is no specific drug to inhibit chromosomal segregation errors in clinical practice (52), proteins regulating the extent of CIN are potential therapeutic targets. For example, the overexpression of *MASTL* can disrupt desmosome function, actin cytoskeleton dynamics, and PI3K/AKT/mTOR and P38 stress kinase signaling pathways, as well as promote CIN in breast cancer (46). By contrast, the inhibition of *MASTL* can induce mitotic disorders and kill breast cancer cells by activating protein phosphatase 2A (66). The protein KIF18A is essential for CIN during the proliferation of cancer cells. Interestingly, the knockout of Kif18A in mice only showed minor defects, indicating that KIF18A may be a safe and an effective therapeutic target. Patients with advanced tumors have been treated with KIF18A inhibitors (40). In addition, *ENPP1* is upregulated in cancer cells with CIN. This protein, which is localized on the cell membrane, can degrade cGAMP, a signaling molecule that can stimulate the immune response. When extracellular cGAMP is degraded, immune cells cannot recognize cancer cells, indicating that it may also be a safe and an effective therapeutic target (67).An interesting new finding is that the chemotherapeutic drug resistance-inducing gene *CKS1B*, a cell cycle progression gene, is closely related to S-CIN and is considered as a novel drug target (25).Lastly, pactinib has clinical significance in breast cancer because it can block the phosphorylation of *IRAK1* and *JAK2* by targeting the 1q21.3 amplification, which feedbacks to improve the sensitivity of cells to pactinib (30).

## 8 Conclusion

CIN drives intra-tumoral heterogeneity, which has a profound impact on the occurrence and development of breast cancer, treatment efficacy, and patient prognosis. With advances in detection technologies, it is possible to gain a deeper understanding of genomic changes and CIN levels in tumors. However, there is a paradoxical relationship between CIN extent, treatment efficacy, and patient prognosis, and further studies are needed to develop new therapeutic strategies aimed at improving the prognosis of breast cancer patients.

## Author contributions

Y-YL finished the review, and W-MC led its drafting and managed the editing of it. All authors contributed to the article and approved the submitted version.

## References

[1] Vishwakarma R McManus KJ. Chromosome instability; implications in cancer development, progression, and clinical outcomes. Cancers (Basel) (2020) 12:e824. doi: 10.3390/cancers12040824 PMC7226245 32235397

[2] Gronroos E López-García C. Tolerance of chromosomal instability in cancer: Mechanisms and therapeutic opportunities. Cancer Res (2018) 78:e6529–35. doi: 10.1158/0008-5472.Can-18-1958 30420473

[3] Bakhoum SF Ngo B Laughney AM Cavallo JA Murphy CJ Ly P. Chromosomal instability drives metastasis through a cytosolic DNA response. Nature. (2018) 553:e467–72. doi: 10.1038/nature25432 PMC5785464 29342134

[4] Bach DH Zhang W Sood AK. Chromosomal instability in tumor initiation and development. Cancer Res (2019) 79:e3995–4002. doi: 10.1158/0008-5472.CAN-18-3235 PMC7694409 31350294

[5] Smid M Hoes M Sieuwerts AM Sleijfer S Zhang Y Wang Y. Patterns and incidence of chromosomal instability and their prognostic relevance in breast cancer subtypes. Breast Cancer Res Treat (2011) 128:e23–30. doi: 10.1007/s10549-010-1026-5 20632083

[6] Tijhuis AE Johnson SC McClelland SE. The emerging links between chromosomal instability (CIN), metastasis, inflammation and tumour immunity. Mol Cytogenet (2019) 12:e17. doi: 10.1186/s13039-019-0429-1 PMC6518824 31114634

[7] Siri SO Martino J Gottifredi V. Structural chromosome instability: Types, origins, consequences,and therapeutic opportunities. Cancers (Basel) (2021) 13:e3056. doi: 10.3390/cancers13123056 PMC8234978 34205328

[8] Novikov NM Zolotaryova SY Gautreau AM Denisov EV. Mutational drivers of cancer cell migration and invasion. Br J Cancer (2021) 124:e102–14. doi: 10.1038/s41416-020-01149-0 PMC7784720 33204027

[9] Duijf PHG Nanayakkara D Nones K Srihari S Kalimutho M Khanna KK. Mechanisms of genomic instability in breast cancer. Trends Mol Med (2019) 25:e595–611. doi: 10.1016/j.molmed.2019.04.004 31078431

[10] Bakhoum SF Kabeche L Murnane JP Zaki BI Compton DA. DNA-Damage response during mitosis induces whole-chromosome missegregation. Cancer Discovery (2014) 4:e1281–9. doi: 10.1158/2159-8290.Cd-14-0403 PMC4221427 25107667

[11] Thompson SL Bakhoum SF Compton DA. Mechanisms of chromosomal instability. Curr Biol (2010) 20:eR285–95. doi: 10.1016/j.cub.2010.01.034 PMC3781365 20334839

[12] Perez de Castro I Malumbres M. Mitotic stress and chromosomal instability in cancer: The case for TPX2. Genes Cancer (2012) 3:e721–30. doi: 10.1177/1947601912473306 PMC3636746 23634259

[13] Hao Z Zhang H Cowell J. Ubiquitin-conjugating enzyme UBE2C: Molecular biology, role in tumorigenesis, and potential as a biomarker. Tumour Biol (2012) 33:e723–30. doi: 10.1007/s13277-011-0291-1 22170434

[14] Rohrberg J Van de Mark D Amouzgar M Lee JV Taileb M Corella A. MYC dysregulates mitosis, revealing cancer vulnerabilities. Cell Rep (2020) 30:e3368–82.e7. doi: 10.1016/j.celrep.2020.02.041 PMC7085414 32160543

[15] Piemonte KM Anstine LJ Keri RA. Centrosome aberrations as drivers of chromosomal instability in breast cancer. Endocrinology. (2021) 162:e208. doi: 10.1210/endocr/bqab208 PMC8557634 34606589

[16] Marteil G Guerrero A Vieira AF de Almeida BP Machado P Mendonça S. Over-elongation of centrioles in cancer promotes centriole amplification and chromosome missegregation. Nat Commun (2018) 9:e1258. doi: 10.1038/s41467-018-03641-x PMC5871873 29593297

[17] Irony-Tur Sinai M Salamon A Stanleigh N Goldberg T Weiss A Wang YH. AT-dinucleotide rich sequences drive fragile site formation. Nucleic Acids Res (2019) 47:e9685–95. doi: 10.1093/nar/gkz689 PMC6765107 31410468

[18] Li S Wu X. Common fragile sites: Protection and repair. Cell Biosci (2020) 10:e29. doi: 10.1186/s13578-020-00392-5 PMC7059258 32166014

[19] Vargas-Rondón N Villegas VE Rondón-Lagos M. The role of chromosomal instability in cancer and therapeutic responses. Cancers (Basel) (2017) 10:e4. doi: 10.3390/cancers10010004 PMC5789354 29283387

[20] Venkatesan S Angelova M Puttick C Zhai H Caswell DR Lu WT. Induction of APOBEC3 exacerbates DNA replication stress and chromosomal instability in early breast and lung cancer evolution. Cancer Discovery (2021) 11:e2456–73. doi: 10.1158/2159-8290.CD-20-0725 PMC8487921 33947663

[21] Ichida K Suzuki K Fukui T Takayama Y Kakizawa N Watanabe F. Overexpression of satellite alpha transcripts leads to chromosomal instability. via segregation errors at specific chromosomes.Int J Oncol (2018) 52:e1685–93. doi: 10.3892/ijo.2018.4321 29568894

[22] Zhang X Kschischo M. Distinct and common features of numerical and structural chromosomal instability across different cancer types. Cancers (Basel) (2022) 14:e1424. doi: 10.3390/cancers14061424 PMC8946057 35326573

[23] Levine MS Holland AJ. The impact of mitotic errors on cell proliferation and tumorigenesis. Genes Dev (2018) 32:e620–38. doi: 10.1101/gad.314351.118 PMC6004076 29802124

[24] Xu Z Verma A Naveed U Bakhoum SF Khosravi P Elemento O. Deep learning predicts chromosomal instability from histopathology images. iScience. (2021) 24:e102394. doi: 10.1016/j.isci.2021.102394 PMC8099498 33997679

[25] Lepage CC Morden CR Palmer MCL Nachtigal MW McManus KJ. Detecting chromosome instability in cancer: Approaches to resolve cell-to-Cell heterogeneity. Cancers (Basel) (2019) 11:e226. doi: 10.3390/cancers11020226 PMC6406658 30781398

[26] Geigl JB Obenauf AC Schwarzbraun T Speicher MR. Defining 'chromosomal instability'. Trends Genet (2008) 24:e64–9. doi: 10.1016/j.tig.2007.11.006 18192061

[27] Zhu L Pan JN Qian Z Ye WW Wang XJ Cao WM. High chromosome instability identified by low-pass whole-genome sequencing assay is associated with TP53 copy loss and worse prognosis in BRCA1 germline mutation breast cancer. Breast Cancer (2022) 29:e103–13. doi: 10.1007/s12282-021-01286-1 PMC8732803 34403063

[28] Dong Z Zhang J Hu P Chen H Xu J Tian Q. Low-pass whole-genome sequencing in clinical cytogenetics: A validated approach. Genet Med (2016) 18:e940–8. doi: 10.1038/gim.2015.199 26820068

[29] Wells D Kaur K Grifo J Glassner M Taylor JC Fragouli E. Clinical utilisation of a rapid low-pass whole genome sequencing technique for the diagnosis of aneuploidy in human embryos prior to implantation. J Med Genet (2014) 51:e553–62. doi: 10.1136/jmedgenet-2014-102497 PMC4112454 25031024

[30] Goh JY Feng M Wang W Oguz G Yatim S Lee PL. Chromosome 1q21.3 amplification is a trackable biomarker and actionable target for breast cancer recurrence. Nat Med (2017) 23:e1319–30. doi: 10.1038/nm.4405 28967919

[31] Stover DG Parsons HA Ha G Freeman SS Barry WT Guo H. Association of cell-free DNA tumor fraction and somatic copy number alterations with survival in metastatic triple-negative breast cancer. J Clin Oncol (2018) 36:e543–53. doi: 10.1200/jco.2017.76.0033 PMC5815405 29298117

[32] Lepage CC Thompson LL Larson B McManus KJ. An automated, single cell quantitative imaging microscopy approach to assess micronucleus formation, genotoxicity and chromosome instability. Cells. (2020) 9:e344. doi: 10.3390/cells9020344 PMC7072510 32024251

[33] Jdey W Thierry S Popova T Stern MH Dutreix M. Micronuclei frequency in tumors is a predictive biomarker for genetic instability and sensitivity to the DNA repair inhibitor AsiDNA. Cancer Res (2017) 77:e4207–16. doi: 10.1158/0008-5472.Can-16-2693 28588010

[34] Kouprina N Liskovykh M Petrov N Larionov V. Human artificial chromosome (HAC) for measuring chromosome instability (CIN) and identification of genes required for proper chromosome transmission. Exp Cell Res (2020) 387:e111805. doi: 10.1016/j.yexcr.2019.111805 PMC7029811 31877307

[35] Ogden A Rida PC Aneja R. Prognostic value of CA20, a score based on centrosome amplification-associated genes, in breast tumors. Sci Rep (2017) 7:e262. doi: 10.1038/s41598-017-00363-w PMC5428291 28325915

[36] Lee K Kim HJ Jang MH Lee S Ahn S Park SY. Centromere 17 copy number gain reflects chromosomal instability in breast cancer. Sci Rep (2019) 9:e17968. doi: 10.1038/s41598-019-54471-w PMC6884473 31784614

[37] Ben-David U Amon A. Context is everything: Aneuploidy in cancer. Nat Rev Genet (2020) 21:e44–62. doi: 10.1038/s41576-019-0171-x 31548659

[38] Santaguida S Amon A. Short- and long-term effects of chromosome mis-segregation and aneuploidy. Nat Rev Mol Cell Biol (2015) 16:e473–85. doi: 10.1038/nrm4025 26204159

[39] Endesfelder D Burrell R Kanu N McGranahan N Howell M Parker PJ. Chromosomal instability selects gene copy-number variants encoding core regulators of proliferation in ER+ breast cancer. Cancer Res (2014) 74:e4853–63. doi: 10.1158/0008-5472.CAN-13-2664 PMC4167338 24970479

[40] Marquis C Fonseca CL Queen KA Wood L Vandal SE Malaby HLH. Chromosomally unstable tumor cells specifically require KIF18A for proliferation. Nat Commun (2021) 12:e1213. doi: 10.1038/s41467-021-21447-2 PMC7900194 33619254

[41] Carter SL Eklund AC Kohane IS Harris LN Szallasi Z. A signature of chromosomal instability inferred from gene expression profiles predicts clinical outcome in multiple human cancers. Nat Genet (2006) 38:e1043–8. doi: 10.1038/ng1861 16921376

[42] Nguyen B Fong C Luthra A Smith SA DiNatale RG Nandakumar S. Genomic characterization of metastatic patterns from prospective clinical sequencing of 25,000 patients. Cell. (2022) 185:e563–75.e11. doi: 10.1016/j.cell.2022.01.003 PMC9147702 35120664

[43] Gao C Su Y Koeman J Haak E Dykema K Essenberg C. Chromosome instability drives phenotypic switching to metastasis. Proc Natl Acad Sci U S A. (2016) 113:e14793–98. doi: 10.1073/pnas.1618215113 PMC5187712 27930335

[44] Cai Y Crowther J Pastor T Abbasi Asbagh L Baietti MF De Troyer M. Loss of chromosome 8p governs tumor progression and drug response by altering lipid metabolism. Cancer Cell (2016) 29:e751–66. doi: 10.1016/j.ccell.2016.04.003 27165746

[45] Lei JT Shao J Zhang J Iglesia M Chan DW Cao J. Functional annotation of ESR1 gene fusions in estrogen receptor-positive breast cancer. Cell Rep (2018) 24:e1434–44.e7. doi: 10.1016/j.celrep.2018.07.009 PMC6171747 30089255

[46] Rogers S McCloy RA Parker BL Gallego-Ortega D Law AMK Chin VT. MASTL overexpression promotes chromosome instability and metastasis in breast cancer. Oncogene. (2018) 37:e4518–33. doi: 10.1038/s41388-018-0295-z PMC6095835 29743597

[47] Donehower LA Soussi T Korkut A Liu Y Schultz A Cardenas M. Integrated analysis of TP53 gene and pathway alterations in the cancer genome atlas. Cell Rep (2019) 28:e3010. doi: 10.1016/j.celrep.2019.08.061 31509758

[48] Rieckhoff J Meyer F Classen S Zielinski A Riepen B Wikman H. Exploiting chromosomal instability of PTEN-deficient triple-negative breast cancer cell lines for the sensitization against PARP1 inhibition in a replication-dependent manner. Cancers (Basel) (2020) 12:e2809. doi: 10.3390/cancers12102809 PMC7601067 33003585

[49] Watkins TBK Lim EL Petkovic M Elizalde S Birkbak NJ Wilson GA. Pervasive chromosomal instability and karyotype order in tumour evolution. Nature. (2020) 587:e126–32. doi: 10.1038/s41586-020-2698-6 PMC7611706 32879494

[50] Mo H Wang X Ma F Qian Z Sun X Yi Z. Genome-wide chromosomal instability by cell-free DNA sequencing predicts survival in patients with metastatic breast cancer. Breast. (2020) 53:e111–18. doi: 10.1016/j.breast.2020.07.004 PMC7503795 32738630

[51] Birkbak NJ Eklund AC Li Q McClelland SE Endesfelder D Tan P. Paradoxical relationship between chromosomal instability and survival outcome in cancer. Cancer Res (2011) 71:e3447–52. doi: 10.1158/0008-5472.CAN-10-3667 PMC3096721 21270108

[52] Bakhoum SF Cantley LC. The multifaceted role of chromosomal instability in cancer and its microenvironment. Cell. (2018) 174:e1347–60. doi: 10.1016/j.cell.2018.08.027 PMC6136429 30193109

[53] Liu Y Xu J Choi HH Han C Fang Y Li Y. Targeting 17q23 amplicon to overcome the resistance to anti-HER2 therapy in HER2+ breast cancer. Nat Commun (2018) 9:e4718. doi: 10.1038/s41467-018-07264-0 PMC6226492 30413718

[54] Zhou R Zhu X Peng Y Zhong L Peng L Yang B. Clinical impact of 11q13.3 amplification on immune cell infiltration and prognosis in breast cancer. Int J Gen Med (2022) 15:e4037–52. doi: 10.2147/IJGM.S360177 PMC9014960 35444456

[55] Jamal-Hanjani M A'Hern R Birkbak NJ Gorman P Gronroos E Ngang S. Extreme chromosomal instability forecasts improved outcome in ER-negative breast cancer: A prospective validation cohort study from the TACT trial. Ann Oncol (2015) 26:e1340–6. doi: 10.1093/annonc/mdv178 26003169

[56] Munro AF Twelves C Thomas JS Cameron DA Bartlett JM. Chromosome instability and benefit from adjuvant anthracyclines in breast cancer. Br J Cancer (2012) 107:e71–4. doi: 10.1038/bjc.2012.232 PMC3389422 22644297

[57] Swanton C Nicke B Schuett M Eklund AC Ng C Li Q. Chromosomal instability determines taxane response. Proc Natl Acad Sci U S A. (2009) 106:e8671–6. doi: 10.1073/pnas.0811835106 PMC2688979 19458043

[58] Bartlett JM Munro AF Dunn JA McConkey C Jordan S Twelves CJ. Predictive markers of anthracycline benefit: A prospectively planned analysis of the UK national epirubicin adjuvant trial (NEAT/BR9601). Lancet Oncol (2010) 11:e266–74. doi: 10.1016/s1470-2045(10)70006-1 20079691

[59] Vargas-Rondón N Pérez-Mora E Villegas VE Rondón-Lagos M. Role of chromosomal instability and clonal heterogeneity in the therapy response of breast cancer cell lines. Cancer Biol Med (2020) 17:e970–85. doi: 10.20892/j.issn.2095-3941.2020.0028 PMC7721098 33299647

[60] Sipos O Tovey H Quist J Haider S Nowinski S Gazinska P. Assessment of structural chromosomal instability phenotypes as biomarkers of carboplatin response in triple negative breast cancer: the TNT trial. Ann Oncol (2021) 32:e58–65. doi: 10.1016/j.annonc.2020.10.475 PMC7784666 33098992

[61] Scribano CM Wan J Esbona K Tucker JB Lasek A Zhou AS. Chromosomal instability sensitizes patient breast tumors to multipolar divisions induced by paclitaxel. Sci Transl Med (2021) 13:eeabd4811. doi: 10.1126/scitranslmed.abd4811 PMC8612166 34516829

[62] Wein L Loi S. Mechanisms of resistance of chemotherapy in early-stage triple negative breast cancer (TNBC). Breast (2017) 34 Suppl 1:eS27–s30. doi: 10.1016/j.breast.2017.06.023 28668293

[63] Lukow DA Sausville EL Suri P Chunduri NK Wieland A Leu J. Chromosomal instability accelerates the evolution of resistance to anti-cancer therapies. Dev Cell (2021) 56:e2427–39.e4. doi: 10.1016/j.devcel.2021.07.009 PMC8933054 34352222

[64] Ippolito MR Martis V Martin S Tijhuis AE Hong C Wardenaar R. Gene copy-number changes and chromosomal instability induced by aneuploidy confer resistance to chemotherapy. Dev Cell (2021) 56:e2440–54.e6. doi: 10.1016/j.devcel.2021.07.006 34352223

[65] Gómez-Miragaya J Díaz-Navarro A Tonda R Beltran S Palomero L Palafox M. Chromosome 12p amplification in triple-Negative/BRCA1-Mutated breast cancer associates with emergence of docetaxel resistance and carboplatin sensitivity. Cancer Res (2019) 79:e4258–70. doi: 10.1158/0008-5472.Can-18-3835 PMC7616973 31213465

[66] Yoon YN Choe MH Jung KY Hwang SG Oh JS Kim JS. MASTL inhibition promotes mitotic catastrophe through PP2A activation to inhibit cancer growth and radioresistance in breast cancer cells. BMC Cancer (2018) 18:e716. doi: 10.1186/s12885-018-4600-6 PMC6034325 29976159

[67] Li J Duran MA Dhanota N Chatila WK Bettigole SE Kwon J. Metastasis and immune evasion from extracellular cGAMP hydrolysis. Cancer Discovery (2021) 11:e1212–27. doi: 10.1158/2159-8290.Cd-20-0387 PMC8102348 33372007

